# Wine Consumption and Lung Cancer Risk: A Systematic Review and Meta-Analysis

**DOI:** 10.3390/nu17081322

**Published:** 2025-04-10

**Authors:** Carlotta Bertola, Camilla Gobbetti, Gaia Baccarini, Roberto Fabiani

**Affiliations:** 1 Section of Hygiene and Public Health, Department of Medicine and Surgery, University of Perugia, 06132 Perugia, Italy; camilla.gobbetti@specializzandi.unipg.it (C.G.); gaia.baccarini@specializzandi.unipg.it (G.B.); 2 Department of Chemistry, Biology and Biotechnology, University of Perugia, 06123 Perugia, Italy; roberto.fabiani@unipg.it

**Keywords:** wine consumption, lung cancer, wine intake, meta-analysis

## Abstract

**Background/Objectives:** Lung cancer is one of the leading causes of cancer-related mortality, with tobacco smoking being the primary risk factor. However, a significant percentage of lung cancer patients are non-smokers, suggesting the involvement of other risk factors, including alcohol consumption. The IARC classifies ethanol as a Group 1 carcinogen, but unlike other alcoholic beverages, wine contains polyphenols with potential health benefits. Some meta-analyses even suggest a protective effect, which led us to conduct our own meta-analysis to further investigate this possible correlation. **Methods:** We conducted a systematic review and stratified the risk across population subgroups based on smoking status and gender. We then performed a categorical “highest vs. lowest” meta-analysis, comparing heavy consumers with very occasional drinkers, using a random-effects model. Only studies examining the risk of developing lung cancer in wine drinkers were included, excluding those with different outcomes, non-primary, ineligible populations, or involving pregnant women. The literature search was conducted in three databases: PubMed, Scopus, and Web of Science. The risk of bias was assessed with the Newcastle–Ottawa quality rating scale for both case–control and cohort studies (NOS), while statistical analyses were performed using the ProMeta 3.0 software. **Results:** The overall analysis showed a non-statistically significant 11% reduction in lung cancer risk (OR = 0.89; 95% CI: 0.77–1.03). The analysis among smokers revealed a significant 22% reduction in lung cancer risk associated with wine consumption (OR = 0.78; 95% CI: 0.62–0.97). However, this effect was lost when the analysis was conducted separately based on the study design. **Conclusions:** No correlation emerged between wine consumption and lung cancer incidence, either in a protective sense or in terms of increased risk. However, further studies are needed to investigate this correlation more accurately, particularly among non-smokers.

## 1. Introduction

In 2022, GLOBOCAN estimated almost 2.5 million new cases of lung cancer (12.4% of total cancer cases) and 1.8 million deaths (18.7% of total cancer deaths), making it the most frequent cancer and cause of cancer death in men and women combined [1]. The disease ranks first among men, while in females, lung cancer is the second most common cancer type and cause of cancer death after breast cancer [1]. It has been observed that there are significant variations in lung cancer incidence and demographic distribution among different countries. Tobacco smoking rates and level of economic development appear to shape these trends. Currently, up to 80% of smokers live in low- or middle-income countries, with over half of lung cancer deaths occurring in less-developed regions. While the majority of cases occur in men, many countries are seeing an increase in lung cancer incidence in women. In fact, lung cancer is now the leading cause of cancer-related deaths among women in several regions, including North America, northwestern Europe, Australia, and New Zealand [2,3].

Smoking is the main risk factor for lung cancer. The excess risk among continuous smokers relative to that among never-smokers is in the order of 20- to 50-fold [4]. However, 15–20% of men diagnosed with lung cancer are non-smokers and the percentage rises to 50%when it comes to women. In Asian women, up to 60–80% of lung cancer diagnoses are not smoke-related [5]. Thus, in non-smokers, other risk factors may play an important role in the onset of the disease. Some environmental risk factors include exposure to secondhand smoke, radon, outdoor/indoor air pollution, and asbestos [6,7]. Additionally, lifestyle factors such as dietary habits may also influence lung cancer risk [8]. A high intake of fruits, vegetables, and fish may have a preventive effect, whereas red meat and processed foods can increase the risk of lung cancer [8]. Among beverages, an increased risk for lung cancer was observed in association with coffee and alcohol intake [9].

According to the World Health Organization, alcohol consumption caused an estimated 2.6 million deaths, including 401,000 cancer deaths, worldwide in 2019 [10]. Excessive consumption of alcoholic beverages is both an individual and societal health problem. It is associated with significant health risks for chronic diseases [11] and is an emerging problem for adolescents and young adults who drink less often but in larger quantities [12]. The biological effects of ethanol are closely related to its metabolism, which involves the conversion into acetaldehyde first and then into acetate [13]. These metabolic pathways release reactive oxygen species (ROS), which notably contribute to alcohol toxicity [13]. Chronic exposure to ethanol is detrimental to the organs of the digestive tract and to both the nervous and cardiovascular systems. Furthermore, there is strong epidemiological evidence that alcohol drinking can cause a dose-related increase in cancer risk in several organs. Clear patterns have initially emerged between alcohol consumption and the development of cancer of the head and neck, esophagus, liver, breast, colon, and rectum [14]. Accordingly, the International Agency for Research on Cancer (IARC) classified alcoholic beverages, for the first time in 1988 and then confirmed the classification in 2012, as carcinogenic to humans (Group 1) [15]. The most recent meta-analysis on the carcinogenicity of ethanol, based on 106 prospective epidemiological studies, has shown a significant dose-dependent increment of the risk for cancer of the esophagus, stomach, liver, pancreas, colon–rectum, larynx, prostate, and breast [16]. In the case of lung cancer, an 11% increment of risk (RR = 1.11, 95% CI: 1.03–1.20) was evident only for heavy drinker men (≥50.0 g/day), whereas in women no significant effect was observed (RR = 0.89, 95% CI: 0.66–1.21) [16]. Based on the above-reported evidence, many institutions and authorities responsible for disease prevention have provided guidelines and recommendations on alcohol consumption [17].

Given that ethanol is a human carcinogen, it is important to underline that its intake can occur through a variety of alcoholic beverages, mainly beer, wine, and distilled spirits. Therefore, the extent of the cancer risk associated with alcohol intake may depend not only on the dose but also on the type of alcoholic beverage consumed. In particular, wine differs from other alcoholic beverages since it is the only one produced by the fermentation of a fruit (crushed grapes) and contains a myriad of bioactive compounds that are not present in other alcoholic beverages [18,19,20]. In addition to alcohol (9–15% by volume), red wine, and to a lesser extent white wine, contains more than 100 polyphenol compounds, including flavonoids, catechins, quercetin, anthocyanins, and stilbenes (resveratrol). These compounds possess potent antioxidant activity and many other cellular effects (regulation of biochemical pathways and gene expression), which may positively affect human health [19,20]. Although there is still considerable ambiguity surrounding wine consumption and health, a recent meta-analysis showed that low to moderate wine consumption has an inverse relationship with cardiovascular mortality, cardiovascular disease, and coronary heart disease [21]. Similarly, intake of wine has been associated with a significantly reduced risk of type 2 diabetes than both beer and spirits intake [22]. The situation is very different with regard to cancer. Some studies have highlighted a clear increase in the risk, albeit with high doses of wine consumption, of esophageal cancer [23], prostate cancer [24], and breast cancer [25].

Regarding the association of wine consumption with lung cancer, the most recent meta-analysis published on the subject dates back to 2007 [26]. In this case, a protective association was observed both when consumption was less than one glass per day (OR = 0.77, 95% CI: 0.59–1.00) and at higher doses (OR = 0.78, 95% CI: 0.60–1.02), although the effect was statistically not significant [26]. The ambivalence of this topic calls for a thorough and up-to-date review of the available evidence to clarify the specific role of wine in the context of lung cancer. Therefore, we conducted an updated meta-analysis to identify gaps in current knowledge and to provide valuable information on possible differences in risk between population subgroups, such as men and women, smokers and non-smokers, or individuals from different geographical areas. Our objective is to assess the relationship between wine consumption and the risk of developing primary lung cancer. Specifically, this meta-analysis aims to determine whether wine consumption is associated with an increased or decreased risk of lung cancer. The results of the present study could suggest directions for future research, contributing to a broader strategy for the prevention and management of lung cancer.

## 2. Materials and Methods

### 2.1. Search Strategy

A systematic review of the available literature was conducted to identify a possible correlation between alcohol intake, specifically wine consumption, and the occurrence of lung cancer across all histological types. This systematic review and meta-analysis is based on Protocol No. CRD42024567352 registered on PROSPERO (https://www.crd.york.ac.uk/prospero/) on 20 July 2024. The study was conducted following the Preferred Reporting Items for Systematic Reviews and Meta-Analysis (PRISMA) guidelines [27]. To gather relevant data and focus exclusively on wine intake, searches were conducted across three databases: PubMed (http://www.ncbi.nlm.nih.gov/pubmed/, accessed on 21 June 2024), Scopus (https://www.scopus.com/, accessed on 21 June 2024), and Web of Science (http://wokinfo.com/, accessed on 21 June 2024). A literature search was conducted using a search string adapted for different settings of the various databases. The string used for the systematic review was the following: (Wine OR “alcohol consumption” OR “alcoholic beverage” OR “alcoholic beverages”) AND (cancer OR tumor OR tumour OR adenoma OR “neoplastic disease” OR neoplasia OR neoplasm) AND lung.

### 2.2. Inclusion and Exclusion Criteria

All the studies discussing the risk of developing lung cancer in wine-drinking subjects were included. Studies that considered outcomes other than those mentioned above, studies that provided no results, non-primary studies, studies with a reference population other than that indicated in the inclusion criteria, and studies involving pregnant women were excluded. To ensure the accuracy of the study, studies that focused generically on alcohol but not on wine were excluded. Inclusion/exclusion criteria were developed according to the Participants, Interventions, Control, Outcomes, Study Design (PICOS) principle, and details are reported in Table S1 in the Supplementary Material.

### 2.3. Selection of Studies and Data Extraction

After importing the articles, screening was conducted using Zotero, a specialized web platform that allows multiple researchers to work remotely simultaneously (https://www.zotero.org/). To ensure the reliability of the selection process, after excluding duplicates, two reviewers (R.F., G.B.) initially screened the articles based on title and abstract analysis. A full-text evaluation of potentially eligible articles was then conducted. The entire process was conducted blinded, and conflicts were resolved by a senior researcher (R.F.).

For data extraction, an Excel spreadsheet (Microsoft Excel 2016) was prepared to organize and collect key information from the studies included in the systematic review for subsequent analysis. Variables extracted included first author, year, [reference], place, study design, name, and population, cases/controls, incident cases, age, follow-up, assessment of wine intake, histologic types, wine consumption categories, OR/RR/HR (95% CI) according to gender/smoking, *p* for trend, and matched or adjusted variables.

### 2.4. Risk of Bias

The risk of bias was assessed with the Newcastle–Ottawa quality assessment scale for case–control and cohort studies (NOS) by two authors (R.F., C.B.), and conflict was resolved by consensus [28]. The Newcastle–Ottawa quality assessment scale criteria included three categories: (1) selection (for both case–control and cohort studies), (2) comparability (for both case–control and cohort studies), (3) exposure (only for case–control studies) and outcome (only for cohort studies). For each of the included studies, the NOS scale is designed for the selection and exposure categories (or selection and outcome categories for cohort studies) to award a maximum of one star for each numbered item, and two stars for the comparability category (for both study designs).

### 2.5. Statistical Analysis

ProMeta 3.0 software (Internovi, Cesena, Italy, 2015) was used for all the analyses. The pooled data for each primary outcome variable and subgroup analyses are presented as relative risk (RR), 95% confidence intervals (95% CI), and forest plots.

Subgroup analyses were performed on (1) smokers/non-smokers and (2) men/women. For the categories of smokers/non-smokers, data about different quantities of smoke (e.g., one pack of cigarettes/day, one pack of cigarettes/week) were reported, when possible, by the authors (G.B, C.B., C.G.).

The first meta-analysis conducted was a categorical meta-analysis examining the risk estimates of highest wine consumption versus lowest. Risk estimates within the same category were combined using the random-effects model across different studies.

### 2.6. Heterogeneity and Publication Bias

Heterogeneity was assessed using the chi-square-based Cochran’s Q statistic. The *I*^2^ values were used to define the level of heterogeneity as follows: no heterogeneity (*I*^2^ = 0–25%), moderate heterogeneity (*I*^2^ = 25–50%), large heterogeneity (*I*^2^ = 50–75%), and extreme heterogeneity (*I*^2^ = 75–100%). Differences with *p* ≤ 0.05 (derived from two-sided statistical tests) were considered statistically significant [29,30].

Publication bias was investigated by the methods of Begg and Mazumdar and Eggers et al. as previously reported [31,32]. The funnel plot asymmetry was tested based on the rank correlation between the effect estimates and their sampling variances, and it was considered asymmetric when the intercept of Egger’s regression line deviated from zero, with a *p*-value ≤ 0.05. The analysis of sensitivity was used to reveal the robustness of combined effect estimates. One study in each turn was omitted to investigate the influence of a single study on the overall risk estimate.

## 3. Results

### 3.1. Study Selection

**Figure 1:**
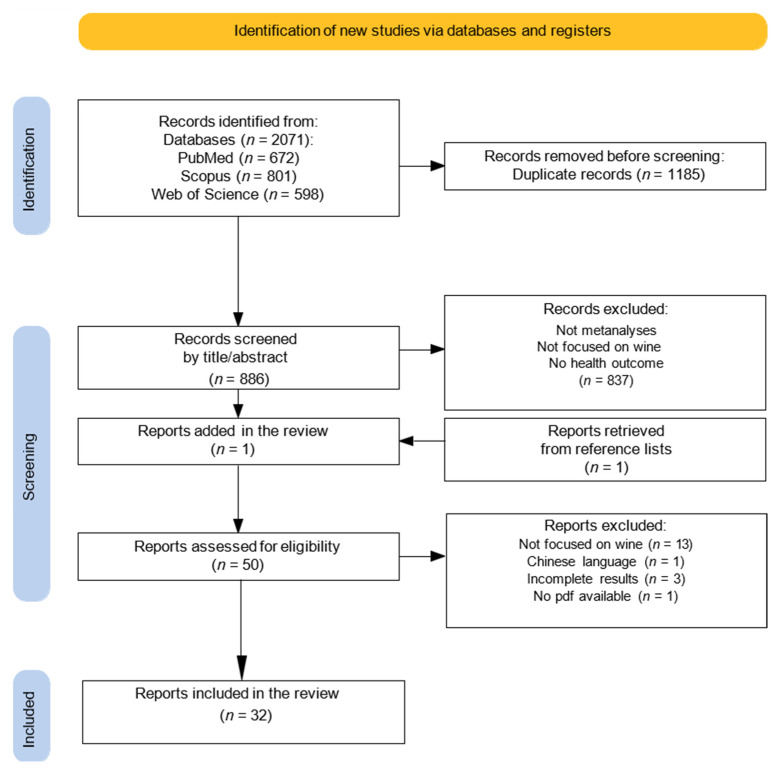
Literature search strategy and study selection.

From the initial search on three different databases (Scopus, Web of Science, PubMed), 2071 articles were identified. After the removal of duplicates (*n* = 1185), 886 articles remained for the title and abstract analysis (Figure 1). After the initial screening, 837 studies were excluded because they did not meet the necessary inclusion criteria. One additional study, retrieved from the latest conducted meta-analysis, was added to the remaining forty-nine studies. After evaluating the full texts of the 50 selected articles, 18 studies were excluded, leaving a total of 32 studies eligible for meta-analysis [33,34,35,36,37,38,39,40,41,42,43,44,45,46,47,48,49,50,51,52,53,54,55,56,57,58,59,60,61,62,63,64] (Figure 1). The list of the excluded articles [65,66,67,68,69,70,71,72,73,74,75,76,77,78,79,80,81,82] with the reasons for exclusion is available in Table S2 of the Supplementary Materials.

### 3.2. Data Extraction and Studies Characteristics

Data extracted were summarized in two tables, divided for study design (one for case–control studies, one for cohort studies) (Table 1 and Table 2). In total, 22 case–control studies [33,34,35,36,37,38,39,40,41,42,43,44,45,46,47,48,49,50,51,52,53,54], ranging between 1989 and 2019, and 10 cohort studies [55,56,57,58,59,60,61,62,63,64], ranging between 1984 and 2019, were included. Of the case–control studies, five were hospital-based [34,35,39,44,53] and seventeen were population-based [33,36,37,38,40,41,42,43,45,46,47,48,49,50,51,52,54], with case numbers ranging from 118 to 19,149, and controls from 141 to 362,340. In cohort studies, the cohort size ranged from 7837 to 492,902 people, and incident cases ranged from 89 to 10,227. The average age range of participants across the 32 included studies was 25 to 90 years, with a few exceptions [40,49,56,58].

Thirteen studies were conducted in Europe [36,38,42,43,44,45,47,48,51,53,56,57,63], nine in the United States [33,34,37,41,49,59,60,61,62], four in Canada [40,46,50,64], two in Uruguay [35,39], and one in Japan [55]. One study conducted in Europe was a polled analysis of six multi-center case–control studies developed in the northwest of Spain [53]. Three additional studies were polled analysis, two of which selected the population from North America, Europe, and Asia within the International Lung Cancer Consortium (ILCCO) and the SYNERGY Consortium [52,54], and the other selected the cohort from the USA, Europe, and Canada [58].

Wine intake was always assessed through questionnaires, often created specifically for the study and not standardized. However, in five instances, semi-structured questionnaires were used [37,40,49,59,60], and in three cases, fully structured questionnaires were employed [38,42,64]. Frequently, patients were asked about their usual wine consumption, with questions covering the past year, the day before the interview, or a specific time point before the interview. Wine was often assessed as a single category, including red, white, and rosé wines, with measures of effect and their relative 95% confidence intervals calculated generically. Occasionally, as in Pollack’s cohort study [55], the category of wine included red, white, and fortified wines, and sake. Overall, only a few studies examined red, white, and rosé wine individually, without considering all types together [45,53,59].

The histologic types of lung cancer were sometimes studied separately and sometimes generically. In the case–control studies, all but two articles presented generic results for all types of lung cancer: two studies provided results for adenocarcinoma and other cancer types, but did not offer cumulative results (expressed as “all types” in Table 1 and Table 2) [39,42]. Three studies [38,48,54] provided results for both specific and cumulative situations. In contrast, all cohort studies presented cumulative results for all histologic types, with two studies [60,62] also offering results for adenocarcinoma and squamous cell carcinoma, and one study defining results for epithelial tumors [55].

In most studies, wine intake was measured in glasses or drinks, equivalent to approximately 125 mL of beverage. In studies where the intake was stated in grams of wine, methods included converting ml of beverage to grams of alcohol was provided, which later will be reported. However, in studies where no clear conversion to grams of alcohol was provided, the drink intake was recorded in terms of frequency. For example, one study measured wine intake in glasses per day over the past year but did not specify the alcohol quantity in grams. The results were reported as originally presented in the study [49]. Two articles reported wine intake in ounces or liters, one of which was a case–control study [41] and the other a cohort study [55].

Whenever possible, the outcome measures reported in Table 1 and Table 2 were expressed according to gender and smoking status. Smoking status was assessed based on current smoking, cigarette pack years, and the number of years since quitting for ex-smokers in most studies. Other frequently adjusted variables included sex, BMI (body mass index), and education level.

When multiple effect measures were available, the OR/RR/HR with the most adjusted variables were reported. Mutual adjustments for other types of beverages (beer, liquor), when available, were highlighted in bold in the “Matched or adjusted variables” column [37,52,54,55,58,59,61].

### 3.3. Quality Assessment

The summary scores from the quality assessment for each study are presented in Table 1 and Table 2, under the NOS column. The score for each domain of all studies included in the systematic review is reported in the Supplementary Materials (Tables S3 and S4 for case–control and cohort studies, respectively). Two researchers independently assessed the quality of each study, and the final result was reached by consensus. For case–control studies, the values of scores ranged from 6 to 8 (median: 7, mean ± SD: 7.2 ± 0.7), while for cohort studies, the scores ranged from 7 to 9 (median: 8, mean ± SD: 8.1 ± 0.8).

The highest score of 9 was obtained in only three cohort studies [62,63,64]. The most common scores were 7 and 8, with more recent studies tending to score higher. Among the case–control studies, the most common flaw was in the selection domain (selection of control subjects), while for cohort studies, it was in the outcome domain (outcome of interest not present at the start of the study). The NOS score for pooled analyses [52,53,54,58] could not be calculated. Details are provided in Tables S3 and S4.

### 3.4. “Highest vs. Lowest” Meta-Analysis

**Figure 2:**
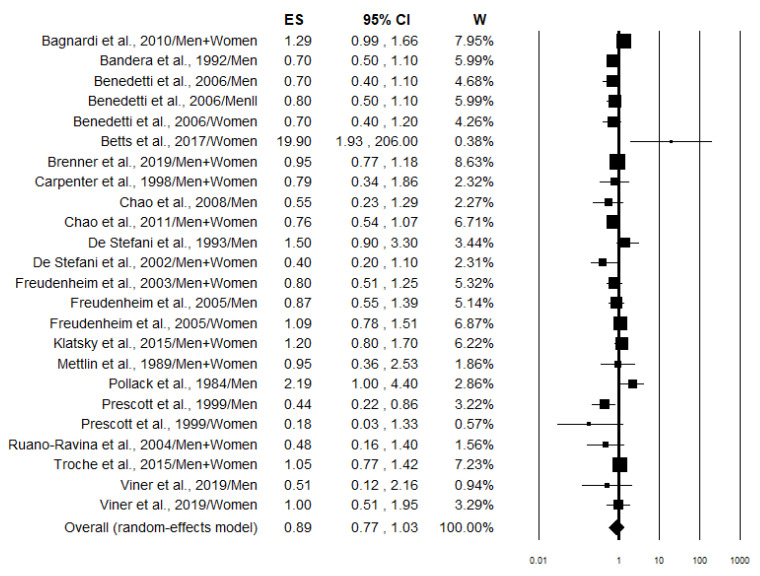
Forest plot showing the pooled analysis [33,34,35,37,39,41,44,46,51,54,55,56,58,59,60,61,63,64] of lung cancer risk associated with the highest wine intake in all subjects (smokers and non-smokers).

**Figure 3:**
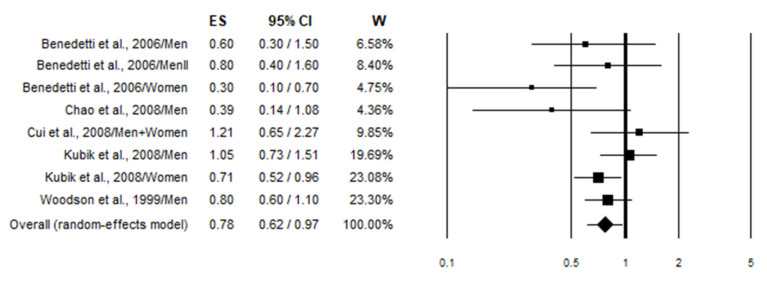
Forest plot showing the pooled analysis [46,48,49,57,59] of lung cancer risk associated with the highest wine intake in smokers.

**Figure 4:**
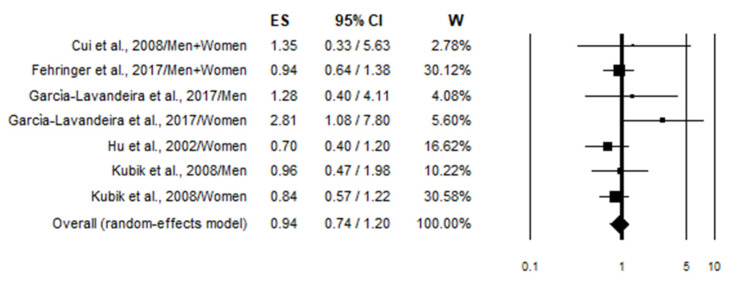
Forest plot showing the pooled analysis [40,48,49,52,53] of lung cancer risk associated with the highest wine intake in non-smokers.

All the studies comparing high wine intake to low were taken into consideration. Studies that provided risk estimates linked to specific types of wine but did not consider all types of wine were excluded from the meta-analysis. Three studies [36,38,50] were excluded for not considering smoking in the adjusted variables, while four articles [42,43,45,49] because they were duplicates of later studies. In total, 25 studies were included in the highest vs. lowest meta-analysis, 15 being case–controls and 10 cohort studies. The overall analysis (combining case–control and cohort studies) of the risk values considering all subjects (smokers and non-smokers) (*n* = 24) showed a statistically non-significant 11% decrease in lung cancer risk in association with wine consumption (OR = 0.89; 95% CI: 0.77–1.03) (Figure 2). Stratified analysis according to the study design produced substantially similar results (Table 3). In particular, case–control studies (*n* = 12), on the basis of 29,506 cases and 388,330 controls, showed a statistically non-significant 14% decrease in lung cancer risk. Meanwhile, cohort studies (*n* = 12), on the basis of a cohort of 1,266,103 subjects and 18,393 incident cases, showed a statistically non-significant 6% decrease in lung cancer risk (Table 3). Instead, the pooled analysis focusing on smokers (*n* = 8) demonstrated a statistically significant 22% reduction in lung cancer risk in association with wine consumption (OR = 0.78; 95% CI: 0.62–0.97) (Figure 3). However, this effect was lost when the analysis was conducted separately in case–control and cohort studies (Table 3). No effect of wine consumption on lung cancer risk was observed even in non-smokers (OR = 0.94; 95% CI: 0.74–1.20) (Figure 4). In this case, no data from cohort studies were available (Table 3). Regarding heterogeneity, it was generally moderate with the *I*^2^ values less than 50% except for cohort studies on all subjects (smokers and non-smokers) in which the *I*^2^ value was found to be 75.4% (Table 3).

A further subgroup analysis based on gender was conducted, and the results are reported in Table S5 in the Supplementary Materials. It is evident that in no case was a statistically significant effect of wine consumption on lung cancer risk observed.

### 3.5. Publication Bias and Sensitivity Analysis

Considering the pooled data and applying both the Egger and Begg tests (Table 3) and funnel plots asymmetry (Figures S1–S3 in the Supplementary Materials), no evident publication bias could be detected for lung cancer risk in smokers and non-smokers (Figure S1), smokers (Figure S2), and non-smokers (Figure S3). Similarly, no publication bias was observed in stratified analysis based on study design (Table 3).

Sensitivity analysis, performed by eliminating each individual study, showed that the lung cancer risk associated with wine consumption was not modified significantly by a single study. In particular, the removal of the outlier study by Betts et al., [63] resulted in an OR of 0.89 (95% CI: 0.77–1.02; *p* = 0.088) in the pooled analysis of all subjects. Similarly, the pooled lung cancer risk estimates calculated in smokers varied from a value of 0.73 (95% CI: 0.57–0.92; *p* = 0.007) when removing the study of Kubik et al. 2008 [48] to 0.82 (95% CI: 0.68–0.98; *p* = 0.030) when omitting the study of Benedetti et al. 2006 [46].

## 4. Discussion

This meta-analysis was conducted to investigate the association between wine consumption (both red and white wine) and lung cancer risk using the “highest versus lowest intake” method (HLM), where the effect size of the highest category of wine consumption is compared to the lowest [83]. After calculating the overall risk, we proceeded to stratify the population according to the smoking status to identify any potential differences. Smoking, in particular, has been shown to have a synergistic effect with alcohol, making it a potential confounding factor [84]. The analysis revealed no association between wine consumption and lung cancer risk in all subjects (Figure 2) and in non-smokers (Figure 4), while a statistically preventive effect was observed in smokers (Figure 3). We performed an additional stratification based on study design, separating cohort studies from case–control studies to identify potential selection and recall biases that typically affect case–control studies (Table 3). Even with this stratification, no association—either positive or negative—with the onset of lung cancer was observed. In fact, the preventive effect of wine on lung cancer in smokers was lost when stratifying by study design (case–control vs. cohort studies), indicating that the result may not be robust despite a *p*-value < 0.05 (Table 3). Further gender stratification did not reveal any significant effect (Table S5).

Our results are partially in agreement with those reported in the previous meta-analysis based on a smaller number of studies (10 case–control and 3 cohorts) in which a statistically significant preventive effect of wine (highest consumption category) was reported in case–control studies (OR = 0.73, 95% CI: 0.63–0.86), but not in cohort studies (OR = 0.95, 95% CI: 0.44–2.04) [26]. Furthermore, the absence of an association between wine consumption and cancer risk has also been observed in previous meta-analyses in the case of bladder [85], ovarian [86], and colorectal cancers [87]. More recently, a systematic review was published that specifically examined the relationship between wine consumption and cancer risk in various body sites [88]. In this work, the authors suggested a potential protective effect for certain cancers, including those of the pancreas, skin, brain, and lungs [88]. However, regarding lung cancer, it is important to note that, in this paper, the authors based their conclusion only on one case–control study from 2006 [46]. Finally, it is worth noting that an additional meta-analysis was published this year, investigating the potential differences between red wine and white wine consumption in relation to cancer in different organs [89]. In the case of lung, only four studies were considered [35,44,59,60] and the pooled analysis showed no differences between lung cancer risk associated with both red (OR = 0.82, 95% CI: 0.41–1.63) and white (OR = 0.79, 95% CI: 0.52–1.22) wine consumption [89].

In an attempt to explain this lack of correlation, one possible factor could lie in the detoxification metabolism of alcohol within the lungs. Lung tissue might be more efficient in breaking down acetaldehyde, reducing lung cell exposure to this metabolite and thereby limiting its carcinogenic potential. [90] Furthermore, the lung contributes to ethanol elimination through respiration, increasing its clearance. [91]. Moreover, other bioactive compounds found in wine (but not in alcohol in general) could also play a role, such as resveratrol and flavonoids. Resveratrol is known for its antioxidant and anti-inflammatory properties, which may help hinder carcinogenesis. Indeed, some experimental studies in animals have shown that resveratrol can modulate pathways involved in oncogenesis by inhibiting oxidative stress, regulating cell proliferation, and promoting the apoptosis of mutated cells [92,93]. Furthermore, resveratrol has been shown to have protective effects against DNA damage and to reduce chronic inflammation, a key factor in tumor development [94]. In addition to resveratrol, wine may contain several other molecules with potential cancer-preventive capacity. In vitro studies have in fact demonstrated that wine, tested as a complete food, is able to inhibit the proliferation and survival of human lung cancer cells, regardless of the presence of resveratrol [95]. Furthermore, it has been shown that the red wine component ellagic acid induces autophagy and exhibits anti-lung cancer activity in vitro and in vivo [96]. Therefore, it is reasonable to assume that the presence of these bioactive molecules in wine can hide and/or inhibit the carcinogenic effect of ethanol.

On the other hand, some other considerations may be useful for interpreting the results of this meta-analysis. Wine drinkers may have healthier lifestyles than nondrinkers or those who drink other alcoholic beverages, especially spirits [97]. For example, some evidence indicate that wine drinkers tend to have a healthier diet. In a Danish population, it was found that wine consumers had higher intakes of fruit, fish, vegetables, and olive oil in comparison to the consumption of other alcoholic beverages [98]. Similarly, wine preference has been strongly associated with healthier eating habits in Western populations [99] and with a higher Healthy Diet Indicator (HDI) score in elderly across Europe [100].

This meta-analysis has certain limitations. Firstly, wine consumption data were derived from self-reported food frequency questionnaires (FFQs), which may introduce recall bias or misreporting. Moreover, not all studies accounted for the consumption of other types of alcohol as a potential confounding variable when assessing the association between wine intake and lung cancer risk. In fact, only six studies (three case–control and three cohort studies) did so (Table 1 and Table 2). Secondly, the analysis does not consider all potential confounding factors affecting lung cancer risks, such as diet, environmental exposures, and genetic predisposition. As a result, the estimated association between wine consumption and lung cancer risk could be biased. Furthermore, the analysis of the included studies revealed a moderate level of heterogeneity, suggesting that differences in study design, population characteristics, or measurement methods may be present. This variability could influence the validity of the meta-analysis results. Another limitation is the absence of a dose–response meta-analysis. Assessing risk based on the “highest vs. lowest” consumption categories compares individuals who drink large amounts of wine with those who consume very little. However, this approach overlooks the full spectrum of consumption patterns and potential trends related to wine intake, potentially leading to an incomplete representation of the association under investigation. The effect size observed in the general population, corresponding to an 11% reduction, was small and did not reach statistical significance. This lack of significance suggests that the observed effect may be attributable to random variability rather than a true impact of the phenomenon under investigation. Consequently, the practical implications of this finding are limited, as there is no strong evidence to support a concrete and generalizable effect across the entire population. Future research should aim to investigate whether wine consumption has different effects on smokers and non-smokers. Additionally, the limited number of studies focusing exclusively on non-smokers prevented a thorough evaluation of potential differences. Most of the available studies either examined only smokers or combined smokers and non-smokers in their analyses, making it difficult to discern potential differences in risk based on smoking status. Conducting studies that stratify participants by smoking status is essential to gaining a clearer understanding of the role of wine consumption in lung cancer risk. Furthermore, the potential presence of other confounding factors in the development of lung cancer beyond smoking, such as diet, genetic predisposition, or environmental exposure, could serve as a valuable starting point for future research. Moreover, investigating the long-term effects of chronic, low-level alcohol consumption could provide further insights into its potential impact on lung cancer risk.

## 5. Conclusions

In conclusion, although alcohol consumption has been shown to be carcinogenic for various body sites and despite several studies considering wine due to its antioxidant substances, our meta-analysis suggests the absence of a correlation between the beverage and the onset of lung cancer. Therefore, we found neither a protective factor nor a risk factor. However, a dose-dependent meta-analysis and new studies, especially on non-smokers, could prove crucial to fully understand the phenomenon.

## Figures and Tables

**Table 1:** Characteristics of the case–control studies included in the systematic revision on the association between wine consumption and lung cancer risk, listed chronologically. Where possible, drinks or glasses have been converted in standard quantities (mL or grams of ethanol).

First Author Year, [Reference] Location	Name Cases/Controls Source of Control Age	Assessment of Wine Intake	Histologic Types	Wine Consumption Categories	OR/HR (95% CI) According to Gender and (Smoking)	*p* for Trend	Matched or Adjusted Variables	NOS
Mettlin et al., 1989 [33] USA	Cases: 569 Controls: 569 Population based Age: 35–90 y range	Questionnaire	All types		Men + Women (All)		Sex, smoking history, beta-carotene intake index and education level	6
				Nondrinkers	1.0 (Ref.)	NA		
				<1 drink/wk	0.55 (0.39–0.79)			
				1–3 drinks/wk	0.51 (0.32–0.82)			
				4–9 drinks/wk	0.82 (0.45–1.50)			
				≥10 drinks/wk	0.95 (0.36–2.53)			
Bandera et al., 1992 [34] USA	Western New York Diet Study Cases: 280 Controls: 564 Population based Age: 35–79 y range	Questionnaire	All types		Men (All)		Age, smoking, education	7
				Nondrinkers	1.0 (Ref.)	0.4		
				1 drink/mo	1.0 (0.7–1.4)			
				≥2 drinks/mo	0.7 (0.5, 1.1)			
De Stefani et al., 1993 [35] Uruguay	Cases: 327 Controls: 350 Hospital based Age: 25–84 y range	Interview	All types		Men (All)		Age, residence, education, and cigarette smoking measured in pack-years	7
				Nondrinkers	1.0 (Ref.)	0.09		
				1–36 mL/day	1.2 (0.70–2.20)			
				37–120 mL/day	1.3 (0.70–3.10)			
				>121 mL/day	1.5 (0.90–3.30)			
Rachtan et al., 1997 [36] Poland	Cases: 118 Controls: 141 Population based Age: NA	Questionnaire	All types		Women (All)		Age	6
				Nondrinkers	1.00 (Ref.)	0.96		
				Rarely	0.90 (0.50–1.81)			
				1–2 drinks/mo	1.08 (0.48–2.45)			
				≥1 drinks/wk	1.16 (0.16–8.45)			
Carpenter et al., 1998 [37] USA	Cases: 261 Controls: 615 Population based Age: 40–84 y range	Semiquantitative food-frequency questionnaire In person interview	All types		Men + Women (All)		Age, race, sex, indicator variables for saturated fat, indicator variables for pack-years, indicator variables for years since quitting smoking and indicator terms for other two alcoholic beverages (all beverage terms in same model).	8
				0–0.42 mL/mo	1.00 (Ref.)			
				0.14–0.84 mL/wk	0.72 (0.41–1.28)			
				≥0.14 mL/day	0.79 (0.34–1.86)			
					In the case of multiple estimates, those that adjusted for the most confounding factors were selected. It was considered the calculation of recent alcohol intake.			
Rachtan, 2002 [38] Poland	Cases: 242 Controls: 352 Population based Age: 61.0 y (median all types) 58.0 y (median controls)	Structured questionnaire	All types		Women (All)		Age	6
				Nondrinkers	1.00 (Ref.)	0.0073		
				Rarely (not specified)	1.29 (0.87–1.93)			
				At least 3 times per month	1.99 (1.19–3.32)			
			Squamous cell		Women (All)			
				Nondrinkers	1.00 (Ref.)	0.4939		
				Rarely (not specified)	1.35 (0.78–2.34)			
				At least 3 times per month	1.09 (0.47–2.51)			
			Adenocarcinoma		Women (All)			
				Nondrinkers	1.00 (Ref.)	0.1971		
				Rarely (not specified)	1.25 (0.62–2.52)			
				At least 3 times per month	1.77 (0.75–4.17)			
			Small cells		Women (All)			
				Nondrinkers	1.00 (Ref.)	0.005		
				Rarely (not specified)	1.34 (0.75–2.38)			
				At least 3 times per month	2.71 (1.40–5.22)			
De Stefani et al., 2002 [39] Uruguay	Cases: 160 Controls: 520 Hospital based Age: 30–89 y range	Questionnaire	Adenocarcinoma		Men + Women (All)		Age, residence, urban/rural status, education, family history of lung cancer in first-degree relatives, body mass index, smoking status, cigarettes per day, years since quit and age at start smoking.	7
				Nondrinkers	1.0 (Ref.)	0.09		
				1–60 mL/day	0.6 (0.3–1.2)			
				61–120 mL/day	0.6 (0.3–1.2)			
				≥121 mL/day	0.4 (0.2–1.1)			
Hu et al., 2002 [40] Canada	NECSS Cases: 161 Controls: 483 Population based Age: 20–70+ y range	Modified block–NCI health habits and history questionnaire	All types		Women (non-smokers)		10-year age groups, province, education, and social class	7
				Nondrinkers	1.0 (Ref.)	0.10		
				≤0.5 drink/wk	0.7 (0.4–1.2)			
				>0.5 drink/wk	0.7 (0.4–1.2)			
Freudenheim et al., 2003 [41] USA	Cases: 168 Controls: 3351 Population based Age: 35–79 y (cases range) 35–65 y (controls range)	Questionnaire	All types	Lifetime alcohol consumption:	Men + Women (All)		Age, education, race, gender, BMI, vegetable intake, fruit intake, total energy intake excluding alcohol, packs smoked per year, years smoked, and an index of passive exposure to smoke at home, work and in other settings.	8
				Nondrinkers	1.00 (Ref.)	0.06		
				≤(~19 L)	0.87 (0.53–1.44)			
				>(~19 L)	0.80 (0.51–1.25)			
				Alcohol consumption in the previous 12–24 mo	Men + Women (All)			
				Nondrinkers	1.00 (Ref.)	0.10		
				≤1.0 L	0.67 (0.36–1.28)			
				>1.0 L	0.72 (0.40–1.29)			
Zatloukal et al., 2003 [42] Czech Republic	Czech Women’s Lung Cancer Study Cases: 366 Controls: 1624 Population based Age: 25–89 y range	Structured questionnaire	Adenocarcinoma		Women (All)		Age, residence, education, and pack-years of smoking	7
				Never	1.0 (Ref.)	0.009		
				Monthly	0.70 (0.44–1.12)			
				Weekly/daily	0.46 (0.23–0.92)			
			Squamous + small + large cells		Women (All)			
				Never	1.0 (Ref.)	0.114		
				Monthly	0.66 (0.43–1.03)			
				Weekly/daily	0.77 (0.47–1.28)			
Kubik et al., 2003 [43] Czech Republic	Cases: 419 Controls: 1593 controls Population based Age: 25–89 y range	Questionnaire	All types		Women (non-smokers)		Age, residence, and education	7
				Monthly or less + weekly or less + daily or less	0.65 (0.41–1.03)	NA		
					Women (Smokers)		Age, residence, education, pack-years of smoking	
				Monthly or less + weekly or less + daily or less	0.69 (0.49–0.98)	NA		
Ruano-Ravina et al., 2004 [44] Spain	Cases: 132 Controls: 187 Hospital based Age: 64.2 (cases mean) 62.5 (controls mean)	Questionnaire	All types		Men + Women (All)		Age, sex, occupation, smoking habit, and total alcohol intake	7
				Nondrinkers	1.00 (Ref.)	NA		
				White wine	1.47 (0.49–4.38)			
				Red wine	0.43 (0.19–0.96)			
				Rosé wine	0.35 (0.09–1.38)			
				All types	0.48 (0.16–1.40)			
				Dose–response analysis (daily number of glasses)	Men + Women (All)		Age, sex, occupation, smoking habit, and total alcohol intake	
				White wine	1.20 (1.01–1.42)			
				Red wine	0.87 (0.77–0.99)			
				Rosé wine	0.97 (0.82–1.14)			
Kubík et al., 2004 [45] Czech Republic	Cases: 435 Controls: 1710 Population based Age: 25–89 y range	Questionnaire	All types		Women (non-smokers)		Age, residence, education	7
				Nondrinkers	1.00 (Ref.)	0.088		
				Monthly (or less)	0.77 (0.46–1.27)			
				Weekly/daily (or less but more than once monthly)	0.52 (0.21–1.27)			
					Women (Smokers)		Age, residence, education, and pack-years of smoking	
				Nondrinkers	1.00 (Ref.)	0.010		
				Monthly (or less)	0.60 (0.39–0.94)			
				Weekly/daily (or less but more than once monthly)	0.60 (0.37–0.98)			
Benedetti et al., 2006 [46] Canada	Cases: † 699 (study I) 1094 (study II) Controls: † 507 (study I) 1468 (study II) Population based Age: 35–70 y range (study I) 35–75 y range (study II)	Questionnaire	All types	STUDY I	Men (All) ±		Age, smoking status, cigarette-year, respondent status, ethnicity, census tract income, years of schooling, and time since quitting	8
				Never weekly	1.00 (Ref.)	0.16		
				1–6 drinks/wk	1.4 (1.0–1.9)			
				≥7 drinks/wk	0.7 (0.4–1.1)			
				STUDY II	Men (All) ±			
				Never weekly	1.00 (Ref.)	0.19		
				1–6 drinks/wk	0.6 (0.4–0.8)			
				≥7 drinks/wk	0.8 (0.5–1.1)			
				STUDY II	Women (All) ±			
				Never weekly	1.0 (Ref.)	0.01		
				1–6 drinks/wk	0.3 (0.2–0.4)			
				≥7 drinks/wk	0.7 (0.4–1.2)			
				STUDY I	Men (Heavy smokers)		Age, respondent status, ethnicity, smoking status, cigarette-years, socio-economic status, years of schooling, and time since quitting	
				Never weekly	1.00 (Ref.)	0.07		
				1–6 drinks/wk	1.9 (1.0–3.8)			
				≥7 drinks/wk	0.6 (0.3–1.5)			
				STUDY II	Men (Heavy smokers)			
				Never weekly	1.00 (Ref.)	0.83		
				1–6 drinks/wk	0.8 (0.5–1.3)			
				≥7 drinks/wk	0.8 (0.4–1.6)			
				STUDY II	Women (Heavy smokers)			
				Never weekly	1.0 (Ref.)	0.27		
				1–6 drinks/wk	0.2 (0.1–0.4)			
				≥7 drinks/wk	0.3 (0.1–0.7)			
Kubìk et al., 2007 [47] Czech Republic	Cases: 569 Controls: 2120 Population based Age: 25–89 y range	Questionnaire	All types		Women (non-smokers)		Age, residence, education and pack-years of smoking	7
				Monthly or less/Weekly or less/Daily or several times per week	0.89 (0.61–1.30)	NA		
					Women (Smokers)			
				Monthly or less/Weekly or less/Daily or several times per week	0.77 (0.58–1.04)	NA		
Kubik et al., 2008 [48] Czech Republic	Cases: 1096 Controls: 2966 Population based Age: 25–89 y range	Questionnaire	All types		Women (non-smokers)		Age, residence, education and pack-years of smoking	7
				Monthly or less/Weekly or less/Daily or several times per week (valid for all the categories) ***	0.84 (0.57–1.22)			
					Women (Smokers)			
				***	0.71 (0.52–0.96)			
					Men (non-smokers)			
				***	0.96 (0.47–1.98)			
					Men (Smokers)			
				***	1.05 (0.73–1.51)			
					Women (NA)		Age, residence, education and pack-years of smoking	
			Adenocarcinoma	***	0.68 (0.49–0.94)			
			Squamous cell	***	0.95 (0.65–1.40)			
			Small cells	***	0.56 (0.36–0.86)			
					Men (NA)			
			Adenocarcinoma	***	0.79 (0.48–1.29)			
			Squamous cell	***	1.00 (0.68–1.48)			
			Small cells	***	0.82 (0.48–1.38)			
Cui et al., 2008, [49] USA	Cases: 558 Controls: 837 Population based Age: 18–65 y range	Semiquantitative Food Frequency Questionnaire (FFQ)	All types	Glasses/day in the past year	Men + Women (Smokers)		Age, sex, race-ethnicity, years of schooling, smoking status, pack-years of tobacco smoking, and daily energy intake (For smokers’ groups) Age, sex, race-ethnicity, years of schooling and daily energy intake (For non-smokers’ group)	8
				0	1.0 (Ref.)	0.029		
				0–1	0.50 (0.34–0.74)			
				>1	0.32 (0.14–0.74)			
					Men + Women (non-smokers)			
				0	1.0 (Ref.)	0.89		
				0–1	1.27 (0.69–2.36)			
				>1	NA			
				Glasses/d life-time average	Men + Women (Smokers)			
				0	1.0 (Ref.)	0.82		
				0–1	0.58 (0.40–0.83)			
				>1	1.21 (0.65–2.27)			
					Men + Women (non-smokers)			
				0	1.0 (Ref.)	0.78		
				0–1	0.81 (0.41–1.59)			
				>1	1.35 (0.33–5.63)			
Benedetti et al., 2009 [50] Canada	Cases: 700 Controls: 507 Population based Age: 35–70 y range	Self-administered questionnaire	All types		Men (All)		Age, years of education, SES (% above global median family income), weekly serving of fruits and vegetables	8
				Never weekly	1.0 (Ref.)	0.66		
				1–6 drinks/wk	1.24 (0.94–1.64)			
				≥7 drinks/wk	0.80 (0.54–1.19)			
Bagnardi et al., 2010 [51] Italy	EAGLE ^1^ Study Cases:1855 Controls: 2065 Population based Age: 35–79 y range	Self-administered questionnaire Type of wine not specified	All types		Men + Women (All)		Sex, age, education, area of residence, packs of cigarettes smoked/d, duration of smoking, time since quitting smoking, other tobacco use, passive smoke exposure, BMI, fruit and vegetable consumption, fresh red and processed meat consumption	8
				Nondrinkers	0.87 (0.62, 1.23)			
				0.1–4.9 g/day	1.0 (Ref.)	0.001 0.009 *		
				5–14.9 g/day	1.03 (0.78–1.35)			
				15–29.9 g/day	1.53 (1.18–1.98)			
				≥30 g/day	1.29 (0.99–1.66)			
Fehringer et al., 2017 [52] North America, Europe, and Asia	ILCCO ^2^ and SYNERGY Project Cases: 2548 Controls: 9362 Population based Both Age: 60.5 (cases mean) 60.8 (controls mean)	Questionnaire	All types		Men + Women (Never-smokers)		Sex, age, ethnicity, education and study center/sub-center. Adjusted for alcohol type (i.e., mutual adjustment for wine, beer, and liquor)	--
				Nondrinkers	1.0 (Ref.)	NA		
				0–4.9 (g/day)	0.80 (0.69–0.94)			
				5–9.9 (g/day)	0.87 (0.69–1.10)			
				10–19.9 (g/day)	0.84 (0.65–1.09)			
				20–29.9 (g/day)	0.62 (0.43–0.89)			
				≥30 (g/day)	0.94 (0.64–1.38)			
García Lavandeira et al., 2017 [53] Spain	Cases: 438 Controls: 863 Hospital based Both Age: 71 (cases median) 66 (controls median)	Questionnaire	All types		Women (Never-smokers)		Age, sex, study, total alcohol intake, education level, living with smokers and radon exposure	--
				Nondrinkers	1.0 (Ref.)	NA		
				Red wine	1.29 (0.79–2.00)			
				White wine	1.16 (0.47–2.14)			
				Rosé wine	0.53 (0.09–2.09)			
				All types	2.81 (1.08–7.80)			
					Men (Never-smokers)			
				Nondrinkers	1.0 (Ref.)	NA		
				Red wine	1.05 (0.42–2.71)			
				White wine	1.69 (0.35–7.51)			
				Rosé wine	--			
				All types	1.28 (0.40–4.11)			
					Men + Women (Never-smokers)			
				Nondrinkers	1.0 (Ref.)	NA		
				Red wine	1.49 (0.93–2.39)			
				White wine	1.52 (0.69–3.33)			
				Rosé wine	0.72 (0.14–2.73)			
				All types	2.62 (1.24–5.62)			
Brenner et al., 2019 [54] North America, Europe and Asia	ILCCO ^2^ and SYNERGY Project Cases: 19,149 Controls: 362,340 Population based Both Age: 61.7 (cases mean) 60.3 (controls mean) ‡	Questionnaire inquiring about lifetime intake	All types		Men + Women (All)		Sex, age, ethnicity, education and study center/sub-center. Adjusted for alcohol type (i.e., mutual adjustment for wine, beer, and liquor)	–
				Nondrinkers	1.0 (Ref.)	NA		
				0–4.9 (g/day)	0.80 (0.71–0.89)			
				5–9.9 (g/day)	0.87 (0.68–1.10)			
				10–19.9 (g/day)	0.88 (0.74–1.04)			
				≥20 (g/day)	0.95 (0.77–1.18)			
			Adenocarcinoma		Men + Women (All)			
				Nondrinkers	1.0 (Ref.)	NA		
				0–4.9 (g/day)	0.81 (0.72–0.90)			
				5–9.9 (g/day)	0.88 (0.68–1.15)			
				10–19.9 (g/day)	0.98 (0.84–1.14)			
				≥20 (g/day)	0.94 (0.78–1.15)			
			Squamous cell		Men + Women (All)			
				Nondrinkers	1.0 (Ref.)	NA		
				0–4.9 (g/day)	0.69 (0.54–0.88)			
				5–9.9 (g/day)	0.67 (0.43–1.03)			
				10–19.9 (g/day)	0.72 (0.54–0.97)			
				≥20 (g/day)	0.84 (0.59–1.19)			

^1^ Environment and Genetics in Lung Cancer Etiology; ^2^ International Lung Cancer Consortium. Legend: BMI, body mass index; CI, confidence interval; HR, hazard Ratio; mo, month; NA, not available; OR, odds ratio; Ref, reference; wk, week; y, years. † Two case–control studies: Study I (men), Study II (men and women). ± For Study I, “Nonsmoker” corresponds to never smoked nearly every day. For Study II, “Nonsmoker” corresponds to never smoked at least once a week. ‡ Time between study enrollment and lung cancer diagnosis (for cases) or the last known date of query (for noncases), but not specified in quantity. *** same category for all the analyses. * Without nondrinkers.

**Table 2:** Characteristics of the cohort studies included in the systematic revision on the association between wine consumption and lung cancer risk, listed chronologically. Where possible, drinks or glasses have been converted in standard quantities (mL, L, or grams of ethanol) following articles’ guidelines.

First Author Year, [Reference] Location	Name and Population Incident Cases Age Follow Up	Assessment of Wine Intake	Histologic Types	Wine Consumption Categories	RR/HR (95% CI) According to Gender and (Smoking)	*p* for Trend	Matched or Adjusted Variables	NOS
Pollack et al., 1984 [55] Japan	Japan–Hawaii Cancer Study Cohort size: 7837 Cases: 89 Age: 50–79 y range Follow up: 14 y	Interview questionnaire –Usual consumption −24 h preceding the interview	Epithelial types		Men (All)		Age, cigarette-smoking status, ability to speak Japanese, ability to read Japanese, birthplace (Japan/USA), diet (Asian/Western), smoking, alcohol content of the other alcoholic beverages	7
				≥50 oz/mo (1.5 L/mo)	2.19 (1.0–4.4)	0.03		
Prescott et al., 1999 [56] Denmark	Copenhagen City Heart Study Cohort size: 28,160 Age: ≥20 y Cases: 674 Follow up: 28 y	Self-administered questionnaire –one point in time	All types		Men (All)		Age, study cohort, smoking and education In the case of multiple estimates, those that adjusted for the most confounding factors were selected.	7
				<12 g/wk	1.00 (Ref.)			
				12–156 g/wk	0.78 (0.63–0.97)			
				>156 g/wk	0.44 (0.22–0.86)			
					Women (All)			
				Nondrinkers	1.00 (Ref.)			
				12–156 g/wk	0.89 (0.59–1.33)			
				>156 g/wk	0.18 (0.03–1.33)			
Woodson et al., 1999 [57] Finland	ATBC ^1^ Study Cohort size: 27,111 Cases: 1059 Age: 50–69 y range Follow up: 7.7 y	Self-administered questionnaire –Previous year	All types		Men (Smokers)		Age, BMI, years smoked, cigarettes/d, and intervention group The RR estimates were unchanged by further adjustment for other alcohol subtypes.	8
				Nondrinkers	1.1 (0.9–1.3)			
				0.09–2 g/day	1.0 (Ref.)	0.02		
				2.1–67.5 g/day	0.8 (0.6–1.1)			
Freudenheim et al., 2005 [58] USA, Netherland, Canada	Pooling Project of Prospective Studies of Diet and Cancer 4 cohorts for men: ATBC ^1^, HPFS ^2^, NLCS ^3^, NYSC ^4^ 5 cohorts for women: CNBSS ^5^, IWHS ^6,^ NLCS ^3^, NYSC ^4^, NHS-A ^7^, NHS-B ^8^ Cohort size: 399,767 Cases: 3137 Age: 15–107 y	Interview questionnaire	All types		Men (All)		Age, education, BMI, energy intake, smoking status and duration. Each analysis is also adjusted for consumption of the other alcoholic beverages	--
				0 (g/day)	1.0 (Ref.)	0.04		
				>0 to <5 (g/day)	0.94 (0.80–1.11)			
				5 to <15 (g/day)	0.66 (0.51–0.87)			
				≥15 (g/day)	0.87 (0.55–1.39)			
					Women (All)			
				0 (g/day)	1.0 (Ref.)	0.99		
				>0 to <5 (g/day)	0.87 (0.72–1.05)			
				5 to <15 (g/day)	0.75 (0.52–1.07)			
				≥15 (g/day)	1.09 (0.78–1.51)			
Chao et al., 2008 [59] USA	^9^ Californian Men’s Health Study: CMHS Cohort size: 84,170 Cases: 210 Age: 45–69 y range Follow up: 3 y (or lung cancer diagnosis, or death)	Semiquantitative food frequency questionnaire	All types	Red wine	Men (All)		Age, white race, annual income, college education, BMI, rigorous physical activity, history of COPD/emphysema, tobacco smoking, dietary intake	8
				Nondrinkers	1.0 (Ref.)	0.06		
				<1 drink/wk	1.15 (0.73–1.81)			
				≥1 drink/wk	0.65 (0.37–1.15)			
				≥1 drink/day	0.55 (0.23–1.29)			
				White wine	Men (All)			
				Nondrinkers	1.0 (Ref.)	0.71		
				<1 drink/wk	0.86 (0.54–1.37)			
				≥1 drink/wk	1.09 (0.62–1.92)			
				≥1 drink/day	0.87 (0.31–2.40)			
				Red wine	Men (Eversmokers)		Age, ethnicity, education, household income, BMI, smoking status (current, and past by quit duration), cigarettes smoked per day, smoking duration, and history of COPD/emphysema. Beer, red wine, white wine, and liquor consumption were mutually adjusted for in the model	
				Nondrinkers	1.0 (Ref.)	0.03		
				<1 drink/wk	1.10 (0.68–1.78)			
				≥1 drink/wk	0.64 (0.35–1.17)			
				≥1 drink/day	0.39 (0.14–1.08)			
				White wine	Men (Eversmokers)			
				Nondrinkers	1.0 (Ref.)	0.71		
				<1 drink/wk	0.83 (0.51–1.35)			
				≥1 drink/wk	0.86 (0.46–1.63)			
				≥1 drink/day	0.94 (0.34–2.62)			
Chao et al., 2011 [60] USA	VITAL ^10^ Study Cohort size: 66,186 Cases: 580 Age: 50–76 y range Follow up: 5–7 y (or death, or lung cancer diagnosis, or withdrawal, or move-out)	Self-administered food frequency questionnaire (FFQ)	All types		Men + Women (All)		Gender, race, education, household income, body mass index, history of COPD/emphysema, cigarette smoking (duration smoked, pack/y, pack/y squared), family history of lung cancer, high intensity physical activity, fat intake, and fruit and vegetable intake	8
				Nondrinkers	1.0 (Ref.)			
				<1 drink/day	0.97 (0.79–1.18)			
				≥1 drink/day	0.76 (0.54–1.07)			
			Adenocarcinoma		Men + Women (All)			
				Nondrinkers	1.0 (Ref.)			
				<1 drink/day	0.95 (0.68–1.32)			
				≥1 drink/day	0.65 (0.37–1.14)			
			Squamous cell		Men + Women (All)			
				Nondrinkers	1.0 (Ref.)			
				<1 drink/day	1.63 (0.99–2.70)			
				≥1 drink/day	1.57 (0.73–3.34)			
Klatsky et al., 2015 [61] USA	Cohorts size: 124,193 Cases: 2672 Age: 41 y (baseline mean) Follow up: 17.8 y mean (max 27–34 y)	Special check-sheet questionnaire	All types		Men + Women (All)		Sex, race/ethnicity, smoking, usual alcohol intake, BMI, level of education, alcoholic beverage preponderance	8
				<1 drink/day	1.0 (Ref.)	>0.05		
				≥3 drinks/day	1.2 (0.80–1.70)			
Troche et al., 2015 [62] USA	NIH-AARP ^11^ Diet and Health Study Cohort size: 492,902 Cases: 10,227 Age: Follow up: 10–11 y	Self-administered baseline questionnaire	All types		Men + Women (All)		Sex, age at baseline, cigarette smoking, pipe and cigar smoking, education, physical activity at work, leisure-time physical activity, energy intake, BMI, race/ethnicity, and Healthy Eating Index-2010 score, which was modified to exclude alcohol. Cigarette smoking status, average number of cigarettes smoked per day, and, for former cigarette smokers, years since cessation	9
				Nondrinkers	1 (Ref.)	NA		
				0.14–6.86 g/day	0.93 (0.89–0.98)			
				7.00–13.86 g/day	0.87 (0.79–0.96)			
				14–41.86 g/day	0.92 (0.84–1.01)			
				≥42 g/day	1.05 (0.77–1.42)			
			Adenocarcinoma		Men + Women (All)			
				Nondrinkers	1 (Ref.)	NA		
				0.14–6.86 g/day	1.02 (0.94–1.10)			
				7.00–13.86 g/day	1.02 (0.88–1.17)			
				14–41.86 g/day	1.06 (0.93–1.22)			
				≥42 g/day	1.42 (0.92–2.18)			
			Squamous cell		Men + Women (All)			
				Nondrinkers	1 (Ref.)	NA		
				0.14–6.86 g/day	0.83 (0.74–0.92)			
				7.00–13.86 g/day	0.73 (0.58–0.92)			
				14–41.86 g/day	0.76 (0.62–0.95)			
				≥42 g/day	0.24 (0.06–0.96)			
			Small cell		Men + Women (All)			
				Nondrinkers	1 (Ref.)	NA		
				0.14–6.86 g/day	0.86 (0.76–0.97)			
				7.00–13.86 g/day	0.80 (0.62–1.04)			
				14–41.86 g/day	0.78 (0.61–1.00)			
				≥42 g/day	0.99 (0.44–2.21)			
			Undifferentiated		Men + Women (All)			
				Nondrinkers	1 (Ref.)	NA		
				0.14–6.86 g/day	0.85 (0.70–1.05)			
				7.00–13.86 g/day	0.75 (0.49–1.14)			
				14–41.86 g/day	0.83 (0.56–1.23)			
				≥42 g/day	2.57 (1.14–5.81)			
Betts et al., 2017 [63] UK	HALS1 ^12^ Cohort size: 8670 Cases: 89 Age: 45 y mean Follow up: 14–15 y (or cancer diagnosis)	Alcohol diary	All types		Men (All)		Ethnicity, income, self-rated health, smoking status, BMI, exercise	9
				Nondrinkers	1.0 (Ref.)	0.916		
				8–112 g/wk †	0.94 (0.38–2.35)			
				120–224 g/wk †	NE			
				>224 g/wk †	NE			
				80 g/wk †	1.03 (0.28–3.71)			
					Women (All)			
				Nondrinkers	1.0 (Ref.)	0.116		
				8–112 g/wk †	0.75 (0.30–1.85)			
				120–224 g/wk †	NE			
				>224 g/wk †	19.9 (1.93–206)	<0.05		
				80 g/wk †	1.37 (0.55–3.42)			
Viner et al., 2019 [64] Canada	ATP ^13^ Cohort size: 26,607 Cases: 199 Age: 36–69 y range Follow up: 16 y max (12.3 y mean)	Canadian Diet History Questionnaire I (CDHQ-I)	All types		Men (All)		Age, sex, marital status, highest level of education, total household income, smoking status, PYs of cigarettes, BMI	9
				Nondrinkers	1.0 (Ref.)	0.38		
				<13.6 g/day	0.88 (0.54, 1.44)			
				≥13.6 g/day	0.51 (0.12, 2.16)			
					Women (All)			
				Nondrinkers	1.0 (Ref)	0.21		
				<13.6 g/day	0.67 (0.46, 0.97)	<0.05		
				≥13.6 g/day	1.00 (0.51, 1.95)			

^1^ Alpha-Tocopherol, Beta-Carotene Cancer Prevention; ^2^ Health Professionals Follow-Up Study; ^3^ Netherlands Cohort Study; ^4^ New York State Cohort; ^5^ Canadian National Breast Screening Study; ^6^ Iowa Women’s Health Study; ^7^ Nurses’ Health Study Section A; ^8^ Nurses’ Health Study Section B; ^9^ California Men’s Health Study; ^10^ VITamins And Lifestyle; ^11^ National Institutes of Health-AARP; ^12^ Health and Lifestyle Survey; ^13^ Alberta’s Tomorrow Project. Legend: BMI, body mass index; CI, confidence interval; COPD, chronic obstructive pulmonary disease; HR, hazard ratio; mo, months; NA, not available; oz, ounces; PY, packs-year; RR; relative risk; wk, week; y, years. † The original article reported the alcohol consumption in units. We converted them in grams using the most recent UK alcohol guidelines (as indicated in the article). * Without nondrinkers.

**Table 3:** Results of stratified analysis according to the study design and smoking status of the lung cancer risk (all types) estimates for the highest compared with the lowest wine intake ^1^.

	No. ^2^	Combined Risk Estimate		Test of Heterogeneity			Publication Bias	
		Value (95% CI)	*p*	Q	*I*^2^%	*p*	*p* (Egger)	*p* (Begg)
Study design								
All studies								
All ^3^	24	0.89 (0.77–1.03)	0.125	44.88	48.76	0.004	0.226	0.244
Smokers	8	**0.78 (0.62–0.97)**	0.028	10.70	34.58	0.152	0.262	0.083
Non-smokers	7	0.94 (0.74–1.20)	0.628	6.67	10.08	0.352	0.138	0.099
Case–control studies								
All	12	0.86 (0.72–1.03)	0.103	18.78	41.43	0.065	0.092	0.373
Smokers	6	0.79 (0.59–1.07)	0.126	8.79	43.12	0.118	0.506	0.188
Non-smokers	7	0.94 (0.74–1.20)	0.628	6.67	10.08	0.352	0.138	0.099
Cohort studies								
All	12	0.94 (0.73–1.21)	0.607	25.82	57.40	0.007	0.805	0.784
Smokers	2	0.66 (0.36–1.23)	0.193	1.75	42.74	0.186	---	---
Non-smokers	0	---	---	---	---	---	---	---

^1^ The risk estimates were calculated using the random-effects model. ^2^ Number of data used to calculate the risk. ^3^ The population consisted of both smokers and non-smokers. In bold are the statistically significant risk values and highlighted in gray, the related *p*-values.

## Data Availability

The data presented in this study are available in this article.

## Supplementary Materials

The following supporting information can be downloaded at: https://www.mdpi.com/article/10.3390/nu17081322/s1, Figure S1. Funnel plot of the pooled analysis of lung cancer risk associated with the highest wine intake in all subjects (smokers and non-smokers); Figure S2. Funnel plot of the pooled analysis of lung cancer risk associated with the highest wine intake in smokers; Figure S3. Funnel plot of the pooled analysis of lung cancer risk associated with the highest wine intake in non-smokers. Table S1. PICOS criteria for inclusion of studies; Table S2. List of studies excluded from the review and specific reason; Table S3. Methodological quality of case–control studies included in the meta-analysis; Table S4. Methodological quality of cohort studies included in the meta-analysis; Table S5. Results of stratified analysis based on gender of the lung cancer risk (all types) estimates for the highest compared with the lowest wine intake.
