# Supplementary material for: Wine Consumption and Lung Cancer Risk: A Systematic Review and Meta-Analysis
Source: Nutrients. 2025 Apr 10;17(8):1322. doi: 10.3390/nu17081322 (PMC12030585; doi:10.3390/nu17081322)
Supplement: Supplementary file 1 [file nutrients-17-01322-s001.zip › nutrients-3557639-supplementary.pdf]

Supplementary Materials: Wine consumption and lung cancer risk: a systematic review and dose-dependent meta-analysis

Table S1. PICOS criteria for inclusion of studies.

**Parameter**	**Criterion**
**P**opulation	Adult population aged 18 or older.
**I**ntervention	Wine intake (i.e., any wine intake, any level or frequency of intake, any type of wine such as red, white, dessert, or not specified) excluding prenatal exposure.
**C**omparison	Adult population aged 18 who were not exposed to wine or had low levels of wine use in systematic reviews on cohort studies.<br>Healthy control or patients with no cancer in systematic reviews on case-control studies.
**O**utcome	Incidence of lung cancer (any type).
**S**tudy design	Case control and cohort studies.

Table S2. List of studies excluded from the review and specific reason.

Reference	Reason of exclusion
1. Dosemeci 1997 [65]	excluded because it speaks generically about alcohol
2. Matos 1998 [66]	excluded because it speaks generically about alcohol
3. Pacella-Norman 2020 [67]	excluded because it speaks generically about alcohol
4. Djousse 2002 [68]	excluded because it speaks generically about alcohol
5. Shi 2004 [69]	excluded because it speaks generically about alcohol
6. Rohrmann 2006 [70]	excluded because it speaks generically about alcohol
7. Bae 2007 [71]	Excluded because it is written in chinese
8. Shimazu 2008 [72]	excluded because it speaks generically about alcohol
9. Ganesh 2011 [73]	excluded because it speaks generically about alcohol
10. Ferreira de Menezes 2015 [74]	excluded because it speaks generically about alcohol
11. Alvarez-Avellòn 2017 [75]	excluded because it speaks generically about alcohol
12. Im 2021 [76]	excluded because it speaks generically about alcohol
13. Rissanen 2021 [77]	excluded because it speaks generically about alcohol
14. Bandera 1997 [78]	excluded because it speaks

	generically about alcohol
15. Williams 1997 [79]	excluded for incomplete results (missing of CI and details about levels of wine exposure not specified)
16. Potter 1992 [80]	excluded for incomplete results
17. Hinds 1980 [81]	excluded for incomplete results
18. Rachtan 2002 [82]	No pdf available

Table S3. Methodological quality of case-control studies included in the meta-analysis *.

First Author, Publication Year [Reference]	Adequate Definition of Cases	Representativeness of Cases	Selection of Control Subjects	Definition of Control Subjects	Control for Important Factor or Additional Factor †	Exposure Assessment	Same Method of Ascertainment for All Subjects	Non Response Rate ‡	Total Quality Scores
Mettlin et al., 1989 [33]	*	*	---	*	**	---	*	---	6
Bandera et al., 1992 [34]	*	*	---	*	**	*	*	---	7
De Stefani et al., 1993 [35]	*	*	---	*	**	*	*	---	7
Rachtan et al., 1997 [36]	*	*	---	*	*	*	*	---	6
Carpenter et al., 1998 [37]	*	*	*	*	**	*	*	---	8
Rachtan, 2002 [38]	*	*	---	*	*	*	*	---	6
De Stefani et al., 2002 [39]	---	*	---	*	**	*	*	*	7
Hu et al., 2002 [40]	*	*	*	*	*	---	*	*	7
Freudenheim et al., 2003 [41]	*	*	*	*	**	*	*	---	8
Zatloukal et al., 2003 [42]	*	*	---	*	**	*	*	---	7
Kubik et al., 2003 [43]	*	*	---	*	**	*	*	---	7
Ruano-Ravina et al., 2004 [44]	*	*	---	*	**	*	*	---	7
Kubík et al., 2004 [45]	*	*	---	*	**	*	*	---	7
Benedetti et al., 2006 [46]	*	*	*	*	**	---	*	*	8

Kubik et al., 2007 [47]	*	*	---	*	**	*	*	---	7
Kubik et al., 2008 [48]	*	*	---	*	**	*	*	---	7
Cui Y. et al., 2008 [49]	*	*	*	*	**	*	*	---	8
Benedetti et al., 2009 [50]	*	*	*	*	**	---	*	*	8
Bagnardi et al., 2010 [51]	*	*	*	*	**	*	*	---	8
Fehringer et al., 2017 [52]									
García Lavandeira et al., 2017 [53]	Being "pooled analysis" of several studies, it is difficult to assign a score								
Brenner et al., 2019 [54]									

* A study could be awarded a maximum of one star for each item except for the item Control for important factor or additional factor. † A maximum of 2 stars could be awarded for this item. Studies that controlled for age received one star, whereas studies that controlled for other important confounders (smoking and total energy intake/BMI) received an additional star. ‡ One star was assigned if there was no significant difference in the response rate between control subjects and cases by using the chi-square test ($p < 0.05$).

Table S4. Methodological quality of cohort studies included in the meta-analysis *.

First Author, Publication Year [Reference]	Representativeness of the Exposed Cohort	Selection of the Unexposed Cohort	Ascertainment of Exposure	Outcome of Interest Not Present at Start of Study	Control for Important Factor or Additional Factor †	Assessment of Outcome	Follow-Up Long Enough for Outcomes to Occur ‡	Adequacy of Follow-Up of Cohorts §	Total Quality Scores
Pollack et al., 1984 [55]	---	*	*	---	**	*	*	*	7
Prescott et al., 1999 [56]	*	*	---	---	**	*	*	*	7
Woodson et al., 1999 [57]	---	*	*	*	**	*	*	*	8
Freudenheim et al., 2005, [58]	Being "pooled analysis" of several studies, it is difficult to assign a score								
Chao et al, 2008 [59]	*	*	*	*	**	*	---	*	8
Chao et al., 2011 [60]	*	*	*	*	**	*	*	---	8
Klatsky et al., 2015 [61]	*	*	---	*	**	*	*	*	8
Troche et al., 2015 [62]	*	*	*	*	**	*	*	*	9
Betts et al., 2017 [63]	*	*	*	*	**	*	*	*	9
Viner et al., 2019 [64]	*	*	*	*	**	*	*	*	9

* A study could be awarded a maximum of one star for each item except for the item Control for important factor or additional factor. † A maximum of 2 stars could be awarded for this item. Studies that controlled for age received one star, whereas studies that controlled for other important confounders (smoking and total energy intake/BMI) received an additional star. ‡ A cohort study with a follow-up time > 6 years was assigned one star. § A cohort study with a follow-up rate > 75% was assigned one star.

Table S5. Results of stratified analysis based on gender of the lung cancer risk (All types) estimates for the highest compared with the lowest wine intake [1.]

		Combined risk estimate		Test of heterogeneity			Publication bias	
	**No.**[2]	**Value (95% CI)**	**p**	**Q**	I^2 **%**	**p**	**p (Egger test)**	**p (Begg test)**
**All studies**								
Men								
All	9	0.82 (0.62-1.08)	0.160	15.57	48.62	0.049	0.886	0.917
Smokers	5	0.83 (0.66-1.04)	0.102	4.40	9.07	0.355	0.196	0.142
Non-smokers	2	1.04 (0.56-1.92)	0.902	0.17	0.00	0.680	---	---
Women								
All	5	0.96 (0.54-1.70)	0.883	11.30	64.60	0.023	0.914	1.000
Smokers	2	0.52 (0.23-1.17)	0.117	2.74	63.50	0.098	---	---
Non-smokers	3	1.02 (0.56-1.84)	0.955	6.02	66.77	0.049	0.421	0.602
Men and Women								
All	10	0.95 (0.80-1.12)	0.539	14.80	39.19	0.097	0.097	0.180
Smokers	1	1.21 (0.65-2.27)	0.550	---	---	---	---	---
Non-smokers	2	0.96 (0.67-1.40)	0.844	0.23	0.00	0.629	---	---
**Case-Control studies**								
Men								
All	4	0.82 (0.62-1.08)	0.162	4.30	30.28	0.231	0.219	0.308
Smokers	3	0.92 (0.69-1.25)	0.605	1.75	0.00	0.417	0.151	0.117
Non-smokers	2	1.04 (0.56-1.92)	0.902	0.17	0.00	0.680	---	---
Women								
All	1	0.70 (0.40-1.20)	0.203	---	---	---	---	---
Smokers	2	0.52 (0.23-1.17)	0.117	2.74	63.50	0.098	---	---
Non-smokers	3	1.02 (0.56-1.84)	0.955	6.02	66.77	0.049	0.421	0.902

Men and Women								
All	7	0.90 (0.69-1.16)	0.409	11.37	47.23	0.078	0.118	0.293
Smokers	1	1.21 (0.65-2.27)	0.550	---	---	---	---	---
Non-smokers	2	0.96 (0.67-1.40)	0.844	0.23	0.00	0.629	---	---
**Cohort studies**								
Men								
All	5	0.79 (0.45-1.39)	0.410	11.26	64.48	0.024	0.773	1.000
Smokers	2	0.66 (0.36-1.23)	0.193	1.75	42.74	0.186	---	---
Non-smokers	0	---	---	---	---	---	---	---
Women								
All	4	1.10 (0.49-2.51)	0.812	9.46	68.30	0.024	0.874	1.000
Smokers	0	---	---	---	---	---	---	---
Non-smokers	0	---	---	---	---	---	---	---
Men and Women								
All	3	0.98 (0.76-1.27)	0.885	3.43	41.66	0.180	0.893	0.602
Smokers	0	---	---	---	---	---	---	---
Non-smokers	0	---	---	---	---	---	---	---

[1]The risk estimates were calculated using the random-effects model. [2]Number of data used to calculate the risk.

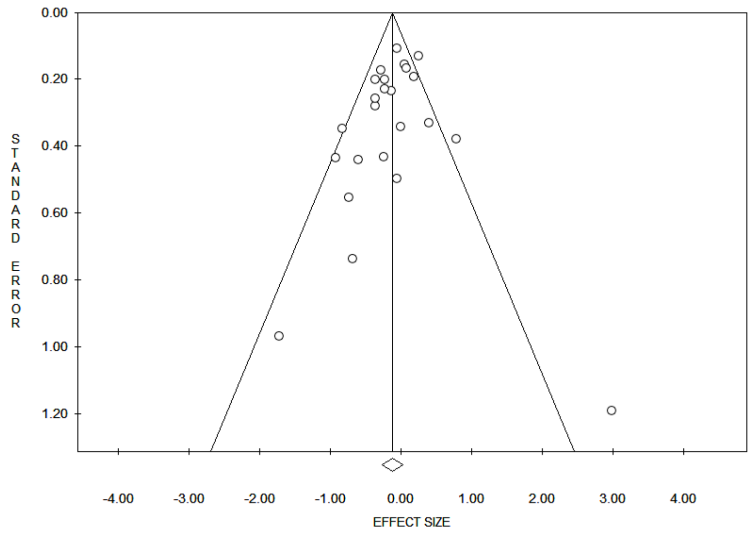

Figure S1. Funnel plot of the pooled analysis [33,34,35,37,39,41,44,46,51,54,55,56,58,59,60,61,63,63,64] of lung cancer risk associated with the highest wine intake in all subjects (smokers and non-smokers).

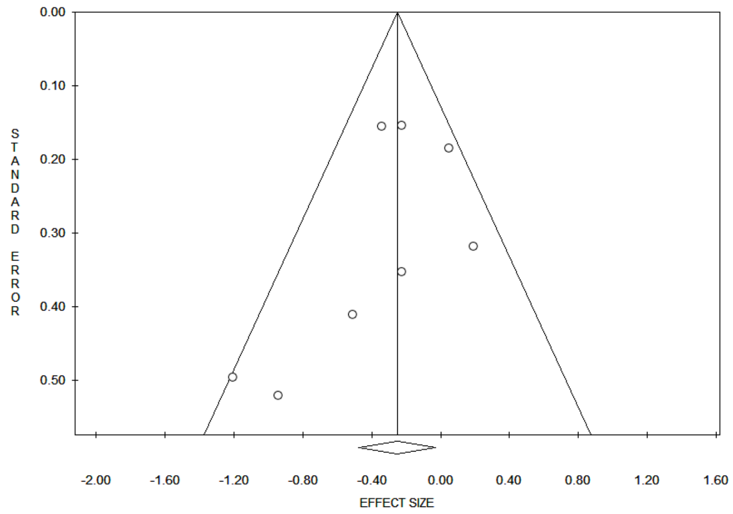

Figures S2. Funnel plot of the pooled analysis [46,48,49,57,59] of lung cancer risk associated with the highest wine intake in smokers.

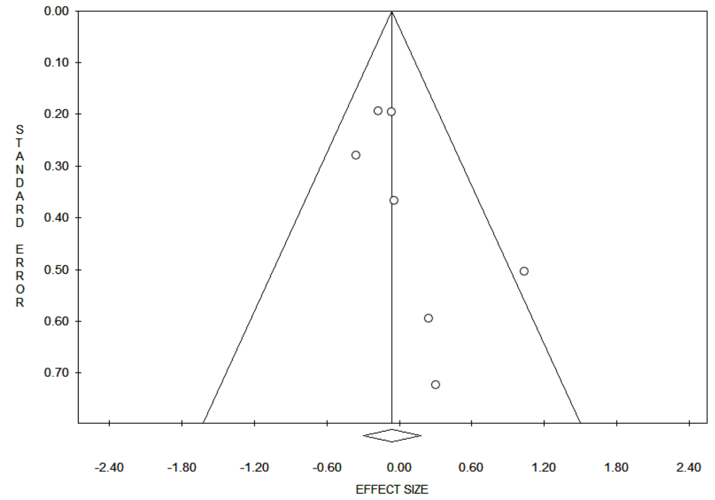

Figure S3. Funnel plot of the pooled analysis [40,48,49,52,53] of lung cancer risk associated with the highest wine intake in non-smokers.
